# Lessons from an elective in Sierra Leone

**DOI:** 10.11604/pamj.2014.17.181.3745

**Published:** 2014-03-07

**Authors:** Tim Robinson

**Affiliations:** 1 College of Medical and Dental Sciences, University of Birmingham, Edgbaston, UK

**Keywords:** Elective, medical schools, Sierra Leone, medical education

## Introduction

Electives are a well-established and popular part of the curriculum of medical schools in Europe and North America, offering students opportunities to expand upon prior learning and gain valuable experience in new environments. In the UK, electives occur after either the fourth or fifth year of undergraduate study, and typically last between six to eight weeks. The majority of students travel abroad, with 40% choosing to go to developing countries [1]. The educational benefits of these placements are varied and far-reaching: aside from improving clinical knowledge and procedural skills through direct exposure to novel scenarios, international health electives can increase students’ awareness of global health issues and their determinants, foster independence and self-confidence, and influence decisions regarding future career choices [2–4]. This article describes my elective in Sierra Leone, particularly focusing on the learning points resulting from this experience.

I should start by answering a question posed countless times by friends, family and colleagues: why Sierra Leone–a country synonymous to most people with danger and insecurity? Firstly, the country's recent history of conflict and the subsequent rebuilding process interested me deeply, and I wanted to see how these devastating events still influenced the lives of people ten years on from the civil war's resolution. Secondly, I wished to experience the delivery of healthcare in a resource-poor setting and the challenges associated with this. Thirdly, I hoped that working in an under-staffed and over-burdened environment would present me with numerous practical learning opportunities. Lastly, I desired to explore the diverse and rich culture that is so unique and fascinating to Africa, and pervades so many aspects of everyday life.

Since the end of the conflict in 2002, which killed 50,000 people and left 2 million displaced [5], Sierra Leone has been making steady progress in a number of areas. Stability is gradually returning to the country, with the 2012 elections being the first in the post-war period to be held without UN supervision. However, Sierra Leone remains one of the least developed nations in the world, ranking 177th out of 186 on the UN Human Development Index [6]. Statistics relating to healthcare are particularly poor: average life expectancy, for example, is 47 years old while the country has just 0.2 physicians per 10,000 population [7]. Nearly 20% of children die before their fifth birthday, with over a third of children under five years being malnourished [7]; while the lifetime risk of maternal death is one in eight and 39% of births lack the presence of a skilled health professional [7, 8].

### Healthcare in Sierra Leone

Sierra Leone's healthcare system comprises a mix of governmental, private and non-governmental organisations (NGOs). There are 13 health districts representing each of the country's administrative areas. A primary care based model, consisting of peripheral health units (PHUs), forms the foundation of healthcare in Sierra Leone; in total there are 1040 PHUs nationwide [9]. In addition, the country has 40 hospitals, of which 23 are state-run [9].

The poor health outcomes among the Sierra Leonean population have been recognised by the government, and in 2010 a scheme to provide free healthcare to pregnant and breastfeeding women and children under the age of five years was launched. The abolishment of user fees among these vulnerable members of society has had promising initial results: the number of children admitted to hospital doubled within the first month of the programme's implementation [1], five times more children are now given the recommended malaria treatment [10], and healthcare seeking behaviours for common childhood illnesses have increased [11]. The number of women delivering in recognised healthcare facilities, as opposed to at home, rose by 45% after the first year of the initiative [12], and overall use of health services in the country is up by 60% [10]. Despite these figures providing much scope for encouragement, the scheme is not without criticism, however. There have been reports of inefficient drug distribution leading to shortages, and people being unnecessarily charged for essential medicines [10, 12]; while poor infrastructure including inadequate transportation systems and lack of electricity and running water have hindered what progress has been made [10]. In addition, questions remain over the programme's sustainability,being heavily reliant on foreign donors whose long-term commitment is uncertain [13, 14]. Nevertheless, these reservations should not cloud what so far appears to be a successful commencement to an ambitious project that has the potential to save tens of thousands of lives.

My elective was based at Magbenteh Community Hospital (MCH) near the town of Makeni, with a population of around 120,000. The hospital was built in 2006 and is run in association with the Swiss-Sierra Leone Development Foundation–a Swiss-based NGO established in 1996. MCH has approximately80 beds on four wards: male, female, maternity and paediatrics. I spent a total of five weeks at MCH;the first two of these were based on the maternity ward, with the remaining three on the male and female wards.

### Learning points from the elective

#### The contrast between theoretical and experiential learning

As a medical student in the UK, much of my learning is done through textbooks; memorising facts so that they may be regurgitated at a later date. Although I try to keep in mind that my present study is for the benefit of the patients I will encounter in my future practice, it is easy to focus on short-term goals – namely passing exams. One reason for going to Sierra Leone was to obtain more practical exposure to those things I had previously learnt, and thus add more depth and longevity to my pre-existing knowledge. With reference to a case I encountered, I will demonstrate how this objective was fulfilled during my elective.

A 42 year-old man, recently diagnosed with type 2 diabetes, was admitted to the male ward with a history of progressive lethargy, weight loss and knee pain. I was able to identify anaemia secondary to diabetic nephropathy as the cause of his lethargy, but was at a loss to explain his knee pain. After one week, his condition deteriorated such that his erythrocyte count was 60 g/l and he was unable to weight-bear on his knee. I sought help from the Chief Medical Officer (CMO), who explained that the knee pain was caused by the patient's poor renal function, which meant he was unable to efficiently filter uric acid; the build-up of which had led to gout. Despite having theoretical knowledge of diabetes, nephrology and rheumatology (all of which were covered during the preceding year of my studies), my relative lack of clinical experience in these areas meant I was unable to put this seemingly unrelated collection of symptoms together to come up with an adequate explanation as to the cause. It is only through being actively involved in this man's care that I have consolidated my existing knowledge of these conditions to an extent that I will now always be able to refer to this case in my future management of patients with type 2 diabetes, mindful of some of the common complications.

I use this case as only one example of such learning: other memorable patients include a young man who developed osteomyelitis after fracturing his ankle falling from a mango tree; a woman with post-partum cardiomyopathy who attended the hospital a month after giving birth suffering with severe orthopnoea; a young lady with known tuberculosis presenting with weakness and paraesthesia of the left leg; and a woman with advanced breast cancer of a severity unimaginable in the UK.

Learning through participation in clinical scenarios such as these has been described to increase students’ self-confidence, motivation and sense of reward, as well as facilitating understanding and knowledge acquisition [15–17]. From my point of view, it was refreshing to learn by ″doing″ rather than ″seeing″. As I look to the final year of my medical training and the two-year Foundation Programme that follows, I am aware that my improving theoretical understanding and growing seniority mean there are sure to be plenty of chances to learn in such a way. Having seen the benefits of experiential learning during my elective, I must ensure I take these valuable opportunities.

#### The practice of medicine in a resource-poor setting

We are extremely lucky in the UK to have a wealth of resources available to shed light on the many questions that emerge during each patient's journey. If someone falls and hits their head, we can perform a CT scan; if someone is jaundiced, we can test their liver function through a simple blood test. Not all countries have these luxuries, however, and healthcare delivery in sub-Saharan Africa is frequently hindered by infrastructure limitations. A review of barriers to emergency and surgical care in five countries describes 22-46% of hospitals having reliable running water and electricity, while 18-41% had adequate storage facilities for medicines [18]. At MCH, basic laboratory tests were available, but more advanced services were hard to come by. An ultrasound machine was functional but unreliable, and the X-ray machine was broken throughout the duration of my placement. Needless to say, these resource limitations sometimes had negative consequences on patient care, as the following example illustrates.

A 24 year-old lady was admitted to the maternity ward after suffering several fits while at home in her village. It was unclear how far advanced she was in her pregnancy, and her notes were contradictory with 28, 34 and 38 weeks all stated. At the time, the ultrasound machine was out of order, so it was not possible to solve this uncertainty. I did, however, speak to two other members of staff involved in the clerking of this patient, who both confirmed that she was 38 weeks’ pregnant. The patient was initially treated with magnesium sulphate, which controlled her seizures. However, in the next few days she developed numerous signs suggestive of serious pre-eclampsia such as a frontal headache, visual disturbances and proteinuria, while her blood pressure peaked at 180/110 mmHg. Despite making every effort to control her condition medically, it soon became clear that the best option for both patient and baby was to perform a caesarean section, and the lady was duly prepared for theatre. She was given a spinal anaesthetic and the team were ready to begin the procedure when the surgeon stated that, due to the doubts over the baby's gestational age, he was unwilling to perform the operation. His examination findings suggested the baby was 34 weeks’ gestation, and given the lack of resources to care for premature babies, would be highly unlikely to survive. Thus, after gathering the appropriate staff, cannulating and catheterising the patient, not to mention putting her through the emotional distress of having to make such an agonising decision to consent to surgery, the procedure was cancelled.

Days later, when the ultrasound machine was once again functional, it emerged that this was the correct course of action as the baby was indeed still several weeks short of full gestation. Thankfully, the patient did not suffer any further complications. Nonetheless, it was extremely frustrating knowing that this situation could have been avoided had a simple investigation, readily available in the UK, been available. However, this is taking a slightly myopic perspective, and one of the many things we as clinicians can learn when working in resource-limited settings is to be more open-minded and creative with our approach to patients. It is easy to become overly reliant on investigations rather than use our intuition, and part of the skill of a doctor is to be able to work around problems, whether relating to the patient or the surrounding environment. Practising medicine in resource-limited settings, far from hindering care, can shift the focus back to the patient, improve empathy and foster a more thoughtful and measured approach to one's work [19–21]. That is certainly true of my experience in Sierra Leone, where I learnt to be more systematic and began to think more analytically about the situations I encountered. By focusing on the basics of good clinical practice–that is to say, taking a thorough history and performing appropriate examinations, I was able to overcome the resource limitations, while at the same time, become a more methodical practitioner and better diagnostician.

#### Adapting to increased levels of responsibility

Aside from the contrasts in learning style and the availability of resources, another factor which struck me during my time in Sierra Leone was the change in my role and, accompanying that, the added responsibility I bore at MCH. My involvement in patient care during my training up to this point had been largely passive, with the onus predominantly being on what I could learn from the case rather than how I could help the patient. Quite rightly, given our lack of qualifications, medical students have a minimal duty of care towards patients and are supervised by senior staff who are ultimately accountable should things go wrong. While I fully understand the reasons for this, in the past I have become frustrated at my lack of involvement in the cases I have encountered, and longed to be able to do more than merely watch. My placement at MCH presented ample opportunities for this void to be filled.

My duties at the hospital closely resembled those I expect to assume when I start working as a doctor. In the mornings I attended the handover meeting, before leading a ward round with community health officers and nurses. Other tasks I would undertake during a typical day included clerking new patients, ordering investigations and interpreting their results, and performing minor procedures such as venepuncture and cannulation. Such a big change in my role, which went from passive observer to proactive doer in a very short space of time, took some getting used to. Although I was able to call upon the CMO for advice, I had the novel and somewhat daunting experience of being responsible for patients.

The relative lack of support, and my elevated status in the hospital when compared to previous experiences in the UK, had both positive and negative aspects. I developed many skills which will be hugely beneficial in my future practice, such as history taking and clinical examination, but also more abstract attributes such as a better judgment, improved decision making and more confidence in my existing knowledge. I particularly enjoyed the patient interaction that my role in the hospital afforded, and, due to my direct involvement in the management of these patients, felt immense satisfaction at seeing positive outcomes. There were times, however, when such responsibility became overwhelming. I occasionally felt out of my depth and doubted my abilities, and several emergency admissions I dealt with were particularly challenging. In addition, I was subjected to the feelings of guilt and regret which accompany the death of a patient under one's care–emotions which become much more intense when one is directly involved as opposed to an onlooker [22]. On these occasions, the assistance of more experienced colleagues would have been invaluable.

Despite these points, I felt it was important to become familiar with the demands of a doctor in anticipation of my imminent Foundation Programme training. The evolution from medical student to doctor is difficult: in 2003, it was reported that over 40% of newly-qualified doctors did not feel their medical school had prepared them adequately [23]. A recent qualitative study showed that medical graduates felt particularly ill-prepared in areas such as ward work, managing acutely ill patients and prescribing [24]. I hope that, despite the vast differences between healthcare in Sierra Leone and the UK, my exposure to some of the common responsibilities I can expect in the coming years of my training will make the transition to being a doctor a smoother one.

#### Ethical issues faced during the elective

It is not uncommon for students partaking in international health electives to encounter challenging ethical scenarios, particularly those students venturing to developing countries. Removed from the comfort and support usually available in their native country, and with altered expectations placed upon them and differing cultural and societal norms, it is perhaps unsurprising that these situations arise. Elit et al. found these issues to manifest in a number of ways. Students reported internal conflict upon exposure to inequalities, and a feeling of being unable to help; unrealistic expectations of their ability and competence by both patients and staff at the host institution; requests to perform procedures beyond their level of training; and guilt that their presence was diverting resources from patients [25].

To a certain extent, these are all points that I can relate to during my elective. Prior to departure, my medical school emphasised the need to maintain the professional values and ethical principles expected of medical students in the UK while on elective. I always bore this advice in mind when I was in Sierra Leone, but nonetheless was perhaps underprepared for some of the situations I faced. As mentioned above, the burden of responsibility on me was a shock initially, but one that I adapted to with time. Despite often working on the wards independently, I always had support from the CMO, and did not hesitate to ask him for help when I felt it was necessary. I ensured I worked within the bounds of my clinical competency; the only occasion this was close to being compromised occurred when I was asked to perform paracentesis on a patient. While I had done this once before under the guidance of the CMO, I stated that I did not feel comfortable doing the procedure unsupervised, and requested the assistance of the CMO. Regarding my role at the hospital, I would like to think that I was more of a help than a hindrance, despite my relative lack of clinical experience. There have (quite rightly) been concerns raised about the sustainability of electives, and questions over whom, out of the student, the patients and the host institution, are the ultimate beneficiaries [26]. Given the scarcity of doctors working at MCH, I felt my input was appreciated by patients and staff alike, although naturally, a longer placement would have been more favourable for all parties.

## Conclusion

Elective placements are highly valued by medical students and for many represent their fondest memories of medical school. This certainly holds true for me: the five weeks I spent in Sierra Leone were challenging, but ultimately enormously enriching and fulfilling. As I had hoped, my elective enabled me to play an active role in patient management and take advantage of practical learning opportunities. I learnt to adapt and work in a setting lacking many of the basic amenities to which I am accustomed, while gaining insight into what it will be like to deal with some of the pressures of being a doctor.

I am extremely glad I followed my conviction to go to a country as unique and fascinating as Sierra Leone, where I was welcomed with sincerity and openness by congenial people. Although I was only there for a short time, “Salone will always occupy a special place in my heart, and I have no doubt that I will become a better doctor because of the experience.
